# Electrical Abnormalities in Dopaminergic Neurons of the Substantia Nigra in Mice With an Aromatic L-Amino Acid Decarboxylase Deficiency

**DOI:** 10.3389/fncel.2019.00009

**Published:** 2019-01-31

**Authors:** Shih-Yin Ho, Yin-Hsiu Chien, Li-Kai Tsai, Shin-ichi Muramatsu, Wuh-Liang Hwu, Horng-Huei Liou, Ni-Chung Lee

**Affiliations:** ^1^Department of Neurology, National Taiwan University Hospital, Taipei, Taiwan; ^2^Department of Pharmacology, College of Medicine, National Taiwan University, Taipei, Taiwan; ^3^Department of Medical Genetics, National Taiwan University Hospital, Taipei, Taiwan; ^4^Department of Pediatrics, National Taiwan University Hospital and National Taiwan University College of Medicine, Taipei, Taiwan; ^5^Division of Neurology, Department of Medicine, Jichi Medical University, Tochigi, Japan; ^6^Center for Gene & Cell Therapy, The Institute of Medical Science, The University of Tokyo, Tokyo, Japan; ^7^Department of Neurology, National Taiwan University Hospital Yunlin Branch, Douliu, Taiwan

**Keywords:** aromatic L-acid decarboxylase, motor disturbances, after-hyperpolarization, substantia nigra, gene therapy, dopaminergic neuron

## Abstract

Aromatic L-acid decarboxylase (AADC) deficiency causes severe motor disturbances in affected children. A putamen-targeted gene therapy improves the motor function of patients. The present study investigated the electrical properties of dopaminergic (DA) neurons in the substantia nigra compacta (SNc) of mice with an AADC deficiency (Ddc^KI^). The basal firing of DA neurons, which determines DA release in the putamen, was abnormal in the Ddc^KI^ mice, including a low frequency and irregular firing pattern, because of a decrease in the after-hyperpolarization (AHP) amplitude of action potentials (APs). The frequency of spontaneous excitatory postsynaptic currents (sEPSCs) increased and that of spontaneous inhibitory PSCs (sIPSCs) decreased in the SNc DA neurons from the Ddc^KI^ mice, suggesting an elevation in glutamatergic excitatory stimuli and a reduction in GABAergic inhibitory stimuli, respectively. Altered expression patterns of genes encoding receptors and channels were also observed in the Ddc^KI^ mice. Administration of a widespread neuron-specific gene therapy to the brains of the Ddc^KI^ mice partially corrected these electric abnormalities. The overexcitability of SNc DA neurons in the presence of generalized dopamine deficiency likely underlies the occurrence of motor disturbances.

## Introduction

Aromatic L-acid decarboxylase (AADC) deficiency (MIM 608643) is a rare genetic disorder caused by a deficiency in AADC activity required for the synthesis of dopamine, serotonin, and other monoamines (Brun et al., 2010). Patients with a severe AADC deficiency experience hypotonia, hypokinesia, dystonia, oculogyric crisis (OGC), and developmental delays because of a dopamine deficiency in the striatum (Brun et al., 2010). Patients also exhibit emotional instability and sleep disturbances because of the serotonin deficiency in the brain and autonomic dysfunction caused by systemic epinephrine and norepinephrine deficiencies (Brun et al., 2010). Due to its rarity, there are no approved treatments for AADC deficiency. We have previously developed a gene therapy for children with AADC deficiency using an intraputaminal injection of adeno-associated virus (AAV) type 2 vectors expressing the *DDC* cDNA (Hwu et al., 2012). Patients exhibited dramatic improvements in motor development and moderate improvements in emotional control and cognitive function after gene therapy (Hwu et al., 2012). However, the underlying cellular mechanism for the motor disturbances of AADC deficiency remains elucidative.

The dopaminergic (DA) system is important for normal motor control. Increase or decrease levels of dopamine in the brain cause a noticeable change in motor performance. A part of the basal ganglia circuit—substantia nigra compacta (SNc) has been found to play a key role in motor function (Schultz, 2007). The SNc contains a large population of DA neuron, which displayed regular/irregular spontaneous firing *in vitro* or *in vivo* (Grace and Bunney, 1984a,b; Grace and Onn, 1989; Cui et al., 2004). Changes in DA neuron firing patterns in SNc may cause the disturbance of information processing in the basal ganglia, resulting in motor-related disorders such as Parkinson’s disease (Bergman et al., 1998). Additionally, the change of firing pattern in mouse models with dopamine deficiency had been described. In DA deficiency mice (*Th*^−/−^; *Dbh*^Th/+^; DD mice), the firing patterns showed only single-spike pattern without bursting activity (Paladini et al., 2003). Furthermore, in Parkinson’s disease-associated mitochondrial dysfunction model with reduced dopamine production (*PINK1-*deficiency mice), the SN DA neurons display hyperexcitability *in vivo* with irregular firing patterns *in vitro* (Bishop et al., 2010). These are considered due to the reduction of endoplasmic reticulum Ca^2+^ release-dependent SK channel function that is mediated by mitochondrial Na^+^/Ca^2+^ exchanger-mediated Ca^2+^ release (Bishop et al., 2010). Because *PINK-1* is predominant in mitochondria (Bishop et al., 2010) and the dysfunction of ER-dependent Ca^2+^ release is explained by mitochondrial dysfunction. We doubt if AADC, the cytosolic enzyme deficiency, underlies the similar mechanism.

The present study investigated the electrical properties of DA neurons in the SNc of a mouse model of AADC deficiency, the Ddc^KI^ mouse. We also administered a widespread neuron-specific gene therapy to Ddc^KI^ mice to confirm the specificity of our findings. The results of this study demonstrated that the alteration of neuronal excitability of SNc DA neurons in the presence of generalized dopamine deficiency likely underlies the occurrence of motor disturbance.

## Materials and Methods

### Animals

AADC-deficient mice (Ddc^KI^) with B6/129 hybrid were maintained by heterozygous mating (Lee et al., 2013). The mice received care according to IACUC guidelines and were maintained on a 12 h light/dark cycle. Wild-type (WT) littermates were used as controls. The mice were sacrificed at 4 weeks of age for electrophysiological analyses, immunofluorescence (IF) staining, or biochemical studies in both genders. Mouse brains used for measurements of DA and serotonin levels and AADC activity were fresh frozen in liquid nitrogen after euthanasia and dissection and stored at −80°C. For IF, the mice were perfused with 4% paraformaldehyde, and the brains were post-fixed overnight at 4°C. Mice were anesthetized by the inhalation of isoflurane inhalation and decapitated as previously described (Ho et al., 2012; Jiang-Xie et al., 2014) to prepare brain slices. Fresh brains were quickly removed into a chilled (0–4°C) cutting solution containing 0.5 mM CaCl_2_, 110 mM choline chloride, 25 mM glucose, 2 mM KCl, 1.25 mM NaH_2_PO_4_, 7 mM MgSO_4_, 26 mM NaHCO_3_, 11.6 mM sodium ascorbate and 3.1 mM sodium pyruvate. Horizontal brain slices (200–220 μm thick) containing SNc neurons were cut using a microslicer (DTK-1000, Dosaka, Kyoto, Japan). Slices were then allowed to recover for at least 1 h prior to recording in artificial cerebrospinal fluid (ACSF) containing 2 mM CaCl_2_, 10 mM glucose, 3 mM KCl, 1 mM MgCl_2_, 125 mM NaCl, 26 mM NaHCO_3_ and 1.25 mM NaH_2_PO_4_ and saturated with 95% O_2_ and 5% CO_2_. For reverse transcription (RT)-PCR analysis, brain sections containing substantia nigra were stored at −20°C with Trizol^®^ before analysis. Total RNA was extracted using the Trizol^®^ reagent and RT were performed according to the manufacturer’s protocol and previous report (Lee et al., 2013).

### Gene Therapy Injection and Neurotransmitter Analyses

A neuron-specific virus (yfAAV9/3-Syn-I-mAADC; AAVN-AADC) with a viral titer of 2.3 × 10^13^ vg/ml was bilaterally injected into the lateral ventricles [intracerebroventricular (ICV) injection; 2 × 10^10^ vg in 2 μl for each hemisphere] of neonatal Ddc^KI^ mice (<24 h; P0) as previously described (Kim et al., 2013; Lee et al., 2014). Analyses of DA and serotonin levels in mouse half brain homogenates were performed by high performance liquid chromatography (HPLC) as previously described methods (Lee et al., 2015). Dual IF staining was performed on 30 μm coronal sections. Tyrosine hydroxylase (TH; 1:800; Millipore, Burlington, MA, USA) and AADC (1:800; a gift from Professor Ichinose, Tokyo Institute of Technology) double staining was also performed, followed by incubations with Alexa Fluor 488-labeled anti-mouse IgG and Alexa Fluor 594-labeled anti-rabbit IgG secondary antibodies (both from Life Technologies, Grand Island, NY, USA). Sections were viewed and photographed using a confocal laser scanning microscope (LSM 510 Meta Confocal Microscope, Zeiss, Germany).

### Electrophysiological Studies

A horizontal single slice was transferred to the recording chamber, held in position using stainless steel grids with a nylon net, and immersed in a continuously flowing solution at a rate of 2–4 ml/min, as previously described (Jiang-Xie et al., 2014). Neurons were visualized under an infrared-DIC camera using an upright microscope (BX51WI, Olympus). Whole-cell recordings were performed at room temperature (22–24°C) using a patch-clamp amplifier (Axopatch 200B, Molecular Devices, San Jose, CA, USA). Patch electrodes (5–8 MΩ) were pulled from borosilicate glass capillaries using a micropipette puller (P97, Sutter Instrument) and filled with a solution containing 140 mM K-Glu, 5 mM KCl, 10 mM HEPES, 0.2 mM EGTA, 2 mM MgCl_2_, 4 mM MgATP, 0.3 mM Na_2_GTP and 10 mM Na_2_-phosphocreatine at pH 7.2 (adjusted with KOH). Data were acquired using a digitizer and pClamp 10 software (Molecular Devices, San Jose, CA, USA). Signals were filtered at 2 kHz and digitized at 10 kHz. SNc DA neurons were characterized by the presence of a large hyperpolarization-activated current (*I*_h_) in response to a hyperpolarizing pulse, as described previously (Grace and Onn, 1989). In some recordings 0.2% w/v biocytin was contained in the internal solution, and conducted immunohistochemistry of TH after experiments. The interspike interval (ISI) and ISI coefficient of variation (CV) were calculated within the first 5 min after the establishment of the whole-cell configuration (current-clamp mode; Dufour et al., 2014). Six previously described action potential (AP) properties were measured: AP threshold, AP rise slope, AP decay slope, AP half width, AP amplitude, and after-hyperpolarization (AHP) amplitude (Dufour et al., 2014). Cells were voltage-clamped at −70 mV in the presence of a GABA receptor antagonist (100 μM picrotoxin) in ACSF to obtain the AMPA receptor-mediated spontaneous excitatory postsynaptic currents (sEPSCs). KCl was substituted for K-Glu, and the cell was voltage-clamped at −70 mV in the presence of NMDA and AMPA receptor antagonists, DL-2-amino-5-phosphonovaleric acid (APV) 50 μM and cyanquixaline (CNQX) 25 μM, respectively, in ACSF to obtain spontaneous inhibitory PSCs (sIPSCs). For investigating the neuronal excitability, 20 μM NMDA was used. Data were acquired using a digitizer and pClamp 10 software (Molecular Devices, San Jose, CA, USA). The signals were filtered at 2 kHz and digitized at 10 kHz. Spontaneous events were detected and analyzed using the MiniAnalysis Program (Synaptosoft Inc.).

### Expression Analysis

Real-time PCR was performed after the reverse transcription of RNA extracted from substantia nigra tissues of WT and Ddc^KI^ mice for gene expression analyses. The PCR results were analyzed using the 2^−ΔΔCT^ method, which represents the quantity of PCR products in Ddc^KI^ mice relative to WT mice after normalization to β-actin.

### Immunofluorescence (IF) Study

Dual IF staining was performed on 30 μm coronal sections. TH (1:800; Millipore) and AADC (1:800; a gift from Professor Ichinose, Tokyo Institute of Technology) double staining was also performed, followed by incubations with Alexa Fluor 488-labeled anti-mouse IgG and Alexa Fluor 594-labeled anti-rabbit IgG secondary antibodies (both from Life Technologies, Grand Island, NY, USA).

### Statistical Analysis

All data are presented as the mean ± SEM, except for the percentage of regular/irregular firing neurons. Statistical analyses were performed using SPSS Statistics Version 17.0 and the Mann-Whitney test for neurotransmitter analysis and expression analysis, Chi-square test for the percentage of irregular firing neurons, *t*-test for electrophysiology analysis, and log-rank (Mantel-Cox) test for survival analysis. A value of *p* < 0.05 was considered significant.

### Ethics Statement

The National Taiwan University College of Medicine and College of Public Health Institutional Animal Care and Use Committee approved this study (IACUC No. 20150435).

## Results

### The Manifestations of Ddc^KI^ Mice Were Corrected by Gene Therapy

**Figure 1:**
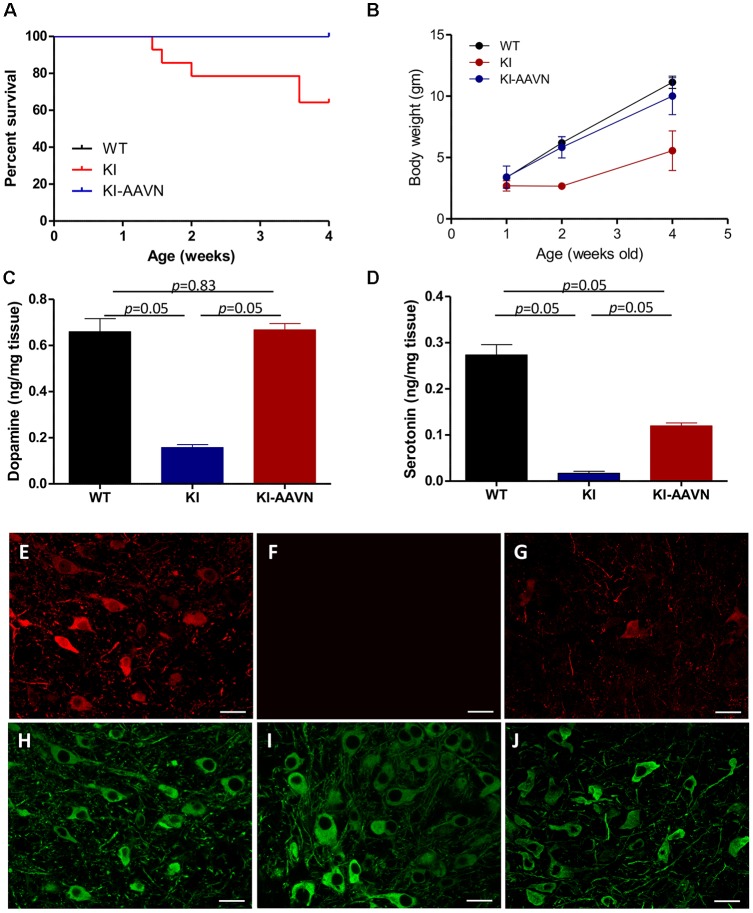
Survival and body weights of the wild-type (WT), Ddc^KI^ (KI), and AAVN-Aromatic L-acid decarboxylase (AADC)-treated KI (KI-AAVN) mice. **(A)** Kaplan-Meier survival curves showing the improved survival of the KI-AAVN mice (WT *n* = 12, KI *n* = 13, KI-AAVN *n* = 12). **(B)** The body weight plot shows that the KI-AAVN mice exhibited catch-up growth at 4 weeks of age (WT *n* = 9, KI *n* = 5, KI-AAVN *n* = 5). **(C)** Dopaminergic (DA) levels in the brains of the WT, KI, and KI-AAVN mice (*n* = 3 each group). The *p* between WT and KI-AAVN is 0.83 (*U* = 4), while *p* between other groups are *p* = 0.05 (*U* = 0). **(D)** Serotonin levels in the brains of the WT, KI, and KI-AAVN mice (*n* = 3 each group). For Figure 1D, p is 0.05 between all three groups while *U* = 0. **(E–J)** Immunofluorescence staining for AADC (red) and TH (green) in coronal brain sections from 4-week-old WT **(E,H)**, KI **(F,I)**, and KI-AAVN **(G,J)** mice.

We previously demonstrated that Ddc^KI^ (KI) mice exhibit a profound DA deficiency, poor weight gain, and early death (Lee et al., 2013); possibly due to reduced food intake and followed by essential compounds deficiency including tryptophan that further worsen the serotonin deficiency; and gene therapy rescues these deficits (Lee et al., 2014, 2015). Gene therapy-treated and untreated mice were used in the present study. The current study administered ICV injections of a neuron-specific vector (yfAAV9/3-Syn-I-mAADC; AAVN-AADC) to mice within 24 h of birth. The AAVN-AADC-treated Ddc^KI^ mice (KI-AAVN mice) exhibited better survival (*p* = 0.023) and higher body weight at 4 weeks of age (*p* = 0.028) than the untreated Ddc^KI^ mice (Figures 1A,B). DA levels in the brains of the treated mice increased to the levels observed in WT mice (*p* = 0.83, *U* = 4, treated vs. WT mice; Figure 1C), and serotonin levels exhibited a 50% increase compared to the levels in WT mice (*p* = 0.05, *U* = 0, treated vs. WT mice; Figure 1D). Double IF staining revealed widespread expression of DDC in neurons throughout the brain, including SNc DA cells (Figures 1E–J).

### Irregular Basal Firing in SNc DA Neurons of Ddc^KI^ Mice

**Figure 2:**
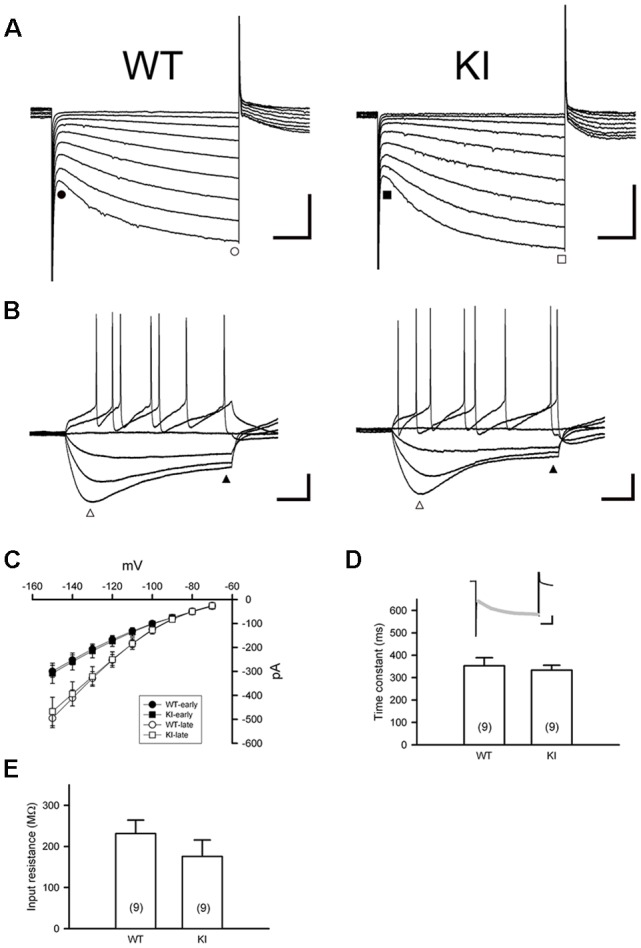
Characterization of *I*_h_ current in substantia nigra DA neurons in the WT and Ddc^KI^ (KI) mice. **(A)** Currents were evoked in individual DA neurons by stepping from a holding potential of −60 mV to membrane potentials ranging from −70 to −150 mV in 10 mV decrements. Scale bars: 200 pA and 200 ms. **(B)** Responses of membrane potential to injection of hyperpolarizing currents (−90 to +60 pA in steps of 25 pA). A prominent inward rectification “sag” occurs during negative current injection (open triangle). Scale bars: 20 mV and 200 ms. **(C)** The current–voltage (I–V) relationships of WT (*n* = 12 cells) and KI mice (*n* = 14 cells). Currents were measured at instantaneous (WT, filled circle; KI, filled square) and steady-state (WT: open circle; KI: open square) during the voltage steps in **(A)**. **(D)** Time constant of activation of *I*_h_ as determined by fitting the activating phase of the current trace with a single exponential function (insert, gray line). Scale bars: 200 pA and 200 ms). No differences were observed between WT and KI mice (*n* = 9 cells for each group). **(E)** Summary bar graph showing the input resistance in WT and KI mice (*n* = 9 cells for each group). Input resistance was calculated from the linear range (−90 to −30 pA) of I–V relationships determined at the end of voltage traces (filled triangle) in (**B**; *n* = 4–5 in each group).

**Figure 3:**
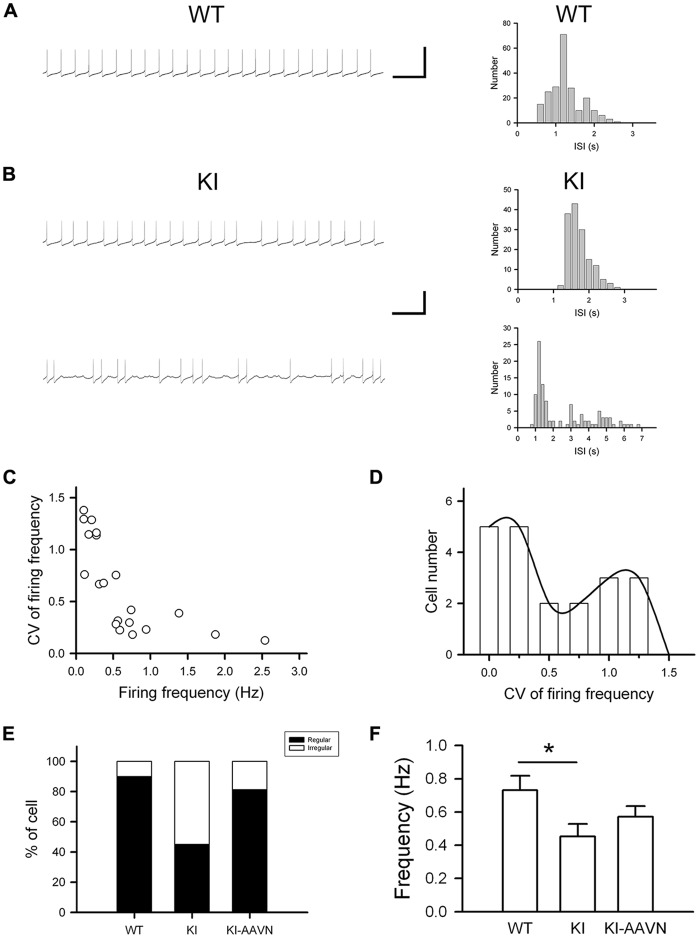
Electrophysiological studies of substantia nigra compacta (SNc) DA neurons in WT, Ddc^KI^ (KI), and AAVN-AADC-treated KI (KI-AAVN) mice. **(A)** Traces of the regular basal firing in neurons from WT mice. The interspike interval (ISI) histogram shows a narrow distribution. Scale bars: 5 s and 100 mV. **(B)** Traces of basal firing in neurons from the KI mice. Some neurons exhibited regular firing patterns similar to the WT mice (upper panel), but other neurons exhibited a slow, irregular firing pattern (lower panel). The ISI histogram of the irregular firing reveals a wide distribution. **(C)** Correlation between the coefficient of variation (CV) of firing frequency and firing frequency for neurons in the KI mice. The CV of slow firing is larger than fast firing. **(D)** Correlation between the CV and cell number in the KI mice. Two populations of neurons with different firing patterns were identified. **(E)** Proportions of the neurons displaying regular and irregular basal firing patterns in the WT (*n* = 11 cells), KI (*n* = 20 cells), and KI-AAVN (*n* = 16 cells) mice. The KI mice exhibited a larger number of irregular firing neurons than the WT mice (*p* = 0.012), and gene therapy partially reversed this abnormality. **(F)** Average firing frequency. A lower frequency was observed in KI mice than in WT mice (*t*_(27)_ = 2.382, *p* = 0.024), and gene therapy partially reversed this abnormality. *p < 0.05.

DA neurons within the SNc fire spontaneously in a highly regular pattern (i.e., tonic or basal firing). We first analyzed the basal firing pattern of SNc DA neurons in Ddc^KI^ mice using whole-cell patch clamp recordings. DA neurons were identified based on their morphology and confirmed by their characteristic *I*_h_ current. Furthermore, in some experiments we filled biocytin in the internal solution and performed TH (a marker for DA neurons) IF staining after recording to confirm DA neurons identity (Supplementary Figure S1). This current was not different between WT and Ddc^KI^ mice (Figures 2A–D). In addition, membrane input resistance is similar in WT and Ddc^KI^ mice DA neurons (Figure 2E). These results indicate that in the absence of AADC, the basic electrophysiological characteristics of DA neurons were identical between Ddc^KI^ and WT mice. DA neurons from WT mice exhibited a regular firing pattern in the recordings (Figure 3A). Some neurons in Ddc^KI^ mice exhibited regular firing, but other neurons exhibited a slow, irregular firing pattern. The ISI histogram revealed a wide distribution (Figure 3B). An analysis of the relationship between firing frequency and the CV revealed that the CV of slow firing neurons was larger than fast firing neurons, indicating that the slow firing was more irregular (Figure 3C). Analysis of the correlations between CV and cell numbers in the Ddc^KI^ mice revealed two populations of neurons with different firing patterns (Figure 3D). We calculated the numbers of regularly or irregularly firing neurons in these mice. The Ddc^KI^ mice displayed a larger number of irregularly firing neurons than the WT mice (*χ*^2^ = 6.304, *p* = 0.012), and gene therapy with AAVN-AADC corrected this abnormality (*χ*^2^ = 4.915, *p* = 0.027; Figure 3E). A lower average firing frequency was observed in the Ddc^KI^ mice than the WT mice (*t*_(27)_ = 2.382, *p* = 0.024), and gene therapy partially corrected this abnormality (Figure 3F). Previous reports have suggested that the *PINK-1* deficient DA neurons also exhibited irregular firing patterns and showed more likely to fire in “NMDA-induced bursting” (Bishop et al., 2010). We therefore examined whether Ddc^KI^ DA neurons also displayed increased neuronal excitability after NMDA application. However, unlike the findings of Bishop et al., our results showed that the Ddc^KI^ DA neurons exhibited similar bursting firing patterns as observed in WT DA neurons (Supplementary Figures S2A,B).

### Abnormalities in APs of SNc DA Neurons in Ddc^KI^ Mice

**Figure 4:**
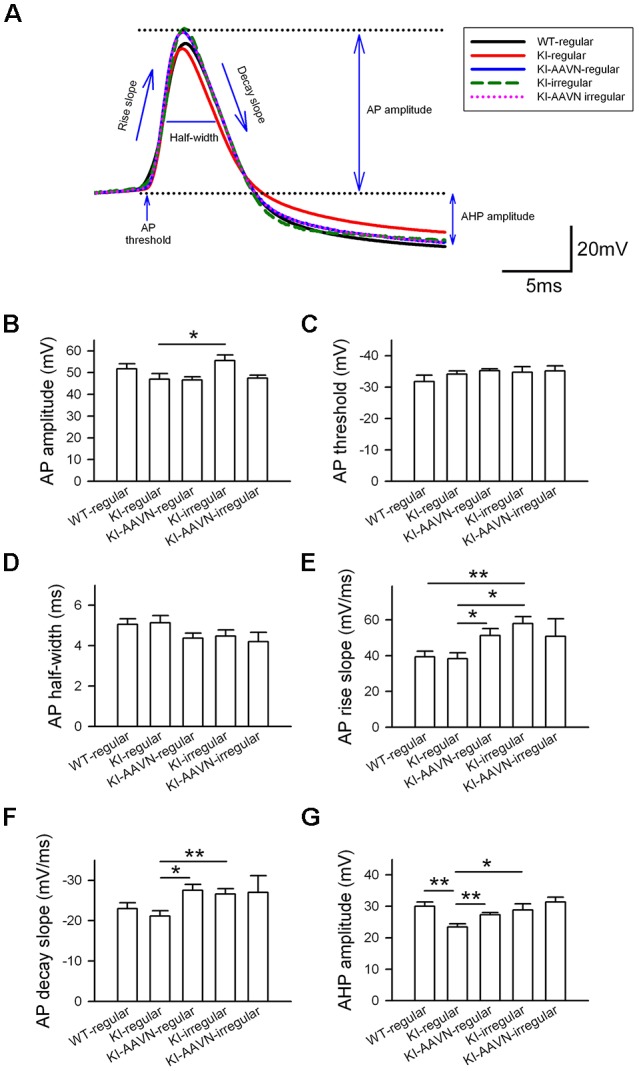
Action potential (AP) properties of SNc DA neurons in WT, KI, and KI-AAVN mice. **(A)** Overlay comparison of APs in WT mice and KI mice. **(B–G)** The APs of regularly firing neurons in KI mice exhibited a smaller after-hyperpolarization (AHP) than WI mice (*p* < 0.01), and the gene therapy with AAVN-AADC corrected this abnormality. The gene therapy with AAVN-AADC also lead to an unexpected increment in AP rise slope and AP decay slope in the Ddc^KI^ regularly firing DA neurons. The comparison of regularly APs in WT mice and the APs of the irregularly firing neurons in KI mice. The APs of the irregularly firing neurons in the KI mice exhibited a faster rise (*p* < 0.05). Furthermore, the irregular firing neurons from Ddc^KI^ mice have higher APs amplitude, rise slope, decay slope and AHP than regular firing neurons. Data were collected from five mice in the WT group, six mice in the KI group and five mice in the KI-AAVN group. *p < 0.05; **p < 0.01.

We then analyzed the AP properties of basal firing using six parameters: amplitude, threshold, half width, rise slope, decay slope, and AHP amplitude (Figure 4A). We first compared the regular APs from the WT mice with the regular APs from the Ddc^KI^ mice and found that the APs of regularly firing neurons in the Ddc^KI^ mice exhibited a smaller AHP than those in the WT mice (*t*_(17)_ = 3.893, *p* = 0.001), and the gene therapy with AAVN-AADC corrected this abnormality (*t*_(20)_ = −3.277, *p* = 0.003, Figure 4G). Unexpectedly, we found the gene therapy with AAVN-AADC result in an increment in APs rise slope and APs decay slope in the Ddc^KI^ regularly firing DA neurons (*t*_(20)_ = −2.439, *p* = 0.024 and *t*_(20)_ = 3.113, *p* = 0.005, respectively, Figures 4E,F). The irregular firing of DA neurons in WT mice is very rare. Therefore, we chose regular firing DA neurons from WT mice to compare with Ddc^KI^ mice irregular firing DA neurons. A comparison of the regularly APs from the WT mice with the APs of the irregularly firing neurons from the Ddc^KI^ mice revealed that the APs of the irregularly firing neurons in Ddc^KI^ mice exhibited a faster rise (*t*_(19)_ = −3.706, *p* = 0.002, Figure 4E). We also compared DA neurons with regular and irregular firing from Ddc^KI^ mice. Our results showed that the irregular firing neurons form Ddc^KI^ mice have higher APs amplitude (*t*_(18)_ = −2.349, *p* = 0.030), rise slope (*t*_(18)_ = −3.774, *p* = 0.001), decay slope (*t*_(18)_ = 3.008, *p* = 0.008) and AHP (*t*_(18)_ = −2.310, *p* = 0.033) than regular firing neurons. However, gene therapy with AAVN-AADC did not correct this abnormality (Figures 4B,E,F,G). No significant differences were detected between WT and Ddc^KI^ SNc DA neurons in APs threshold and half-width (Figures 4C,D).

### Abnormalities in Postsynaptic Currents in SNc DA Neurons of Ddc^KI^ Mice

**Figure 5:**
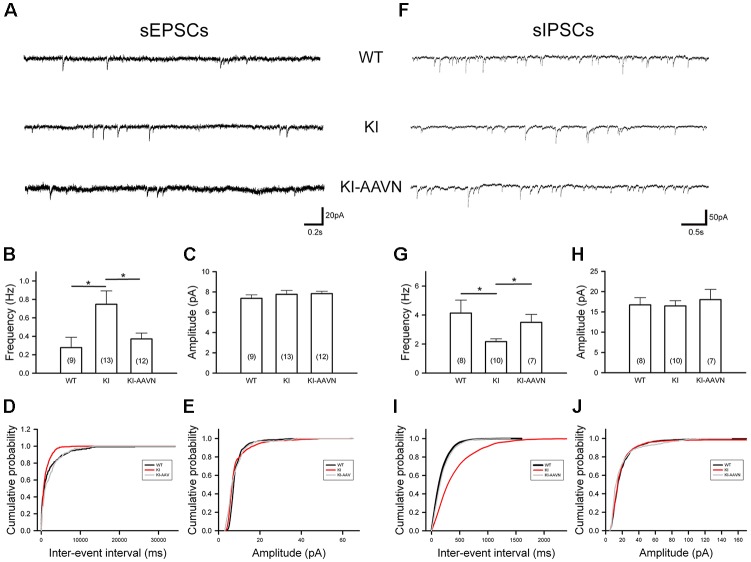
Spontaneous excitatory postsynaptic currents (sEPSCs) and spontaneous inhibitory PSCs (sIPSCs) recorded from SNc DA neurons in WT (*n* = 8–9 cells), KI (*n* = 10–13 cells), and AAVN-AADC-treated KI (KI-AAVN, *n* = 7–12 cells) mice. **(A)** Representative sEPSC traces. **(B)** Calculated average frequency of sEPSCs. A higher frequency was observed in KI mice, and gene therapy corrected this abnormality. Scale bars: 0.2 s and 20 pA. **(C)** The calculated average amplitude of sEPSCs was not different between the three groups of mice. **(D)** A cumulative probability plot of inter-event intervals revealed a smaller interval in KI mice. **(E)** The cumulative probability plot of amplitudes revealed no difference between the three groups. **(F)** Representative sIPSC traces. Scale bars: 0.5 s and 50 pA. **(G)** Calculated average frequency of sIPSCs. The frequency was lower in KI mice than in WT mice, and gene therapy corrected this abnormality. **(H)** The calculated average amplitudes of sIPSCs were not different between the two groups. **(I)** The cumulative probability plot of inter-event intervals revealed a larger interval in KI mice. **(J)** The cumulative probability plot of amplitudes revealed no difference between the two groups. Data are presented as the mean ± SEM, *p < 0.05, *n* = 4–6 in each group.

Synaptic currents from glutamatergic and GABAergic afferents modulate the firing of SNc DA neurons. We recorded sEPSCs of SNc DA neurons (Figure 5A). The average frequency of sEPSCs was higher in the Ddc^KI^ mice than in the WT mice (*t*_(20)_ = −2.383, *p* = 0.027), and gene therapy corrected this abnormality (*t*_(23)_ = 2.318, *p* = 0.030 for the comparison between untreated and treated Ddc^KI^ mice; Figure 5B). The amplitude of sEPSCs did not differ between the groups, which excludes the effects of postsynaptic regulation (Figure 5C). Similar conclusions were obtained from the cumulative probability plot (Figures 5D,E). We also recorded sIPSCs of SNc DA neurons (Figure 5F). The average frequency of sIPSCs was lower in the Ddc^KI^ mice than in the WT mice (*t*_(16)_ = 2.378, *p* = 0.030), and gene therapy corrected this abnormality (*t*_(15)_ = −2.583, *p* = 0.021 for the comparison between untreated and treated Ddc^KI^ mice; Figure 5G). The amplitude of sIPSCs did not differ between the two groups, which excludes the effect of post-synaptic regulation (Figure 5H). Similar conclusions were obtained from the cumulative probability plot (Figures 5I,J).

Real-time PCR analyses revealed a general increase in the expression of genes encoding the glutamate and GABA receptors. Although none of these increases were statistically significant, the expression levels of *Kcnn3* decreased (*p* = 0.05) while the levels of *Gabra3* increased (*p* = 0.05; Supplementary Table S1).

## Discussion

### Abnormalities in the Electrical Activity of SNc DA Neurons in Ddc^KI^ Mice

In the present study, the SNc DA neurons in Ddc^KI^ mice were divided into two groups based on basal firing patterns: regular and irregular/slow firing. The AHP amplitude of regular basal firing DA neurons was low. Additionally, the frequency of sEPSCs increased and that of sIPSCs decreased in the SNc DA neurons from the Ddc^KI^ mice, suggesting an elevation in glutamatergic excitatory stimuli and a reduction in GABAergic inhibitory stimuli, respectively. The administration of a widespread gene therapy to the brain corrected these electric abnormalities, confirming the specificity of these findings. These electric abnormalities in SNc DA neurons likely underlie the occurrence of motor disturbances in patients with an AADC deficiency.

### External Stimulation of SNc DA Neurons From Ddc^KI^ Mice

SNc DA neurons are known to receive several hundred monosynaptic inputs from different brain regions (Watabe-Uchida et al., 2012). Although DA neurons lost their afferent activity in *ex vivo* brain slice. Previous studies revealed that occasionally single quantal release of glutamate or GABA could influence the interneuron regular firing in cerebellar slices (Carter and Regehr, 2002). Furthermore, spontaneous glutamatergic or GABAergic neurotransmission has been shown to regulate DA neurons firing (Xiao et al., 2009; Xie et al., 2012). In the present study, we observed an increase in spontaneous glutamatergic neurotransmission for the SNc DA neurons from the Ddc^KI^ mice. Glutamatergic afferents to the SNc primarily arise from the subthalamic nucleus, prefrontal cortex, and pedunculopontine nucleus (Watabe-Uchida et al., 2012; Zakharov et al., 2016), and hyperactivation of the motor cortex was described in patients with PD and animal models of PD (Bezard et al., 1997; Yu et al., 2007). Approximately 70% of the afferents to SNc DA neurons are GABAergic, and most of these afferents arise from the neostriatum, the external globus pallidus, and the substantia nigra pars reticulata (SNr). Tonic inhibitory signals from GABAergic afferents (primarily mediated through GABA_A_ receptors) prevent burst firing (Paladini and Tepper, 2017). Our data revealed a significant decrease in spontaneous GABAergic neurotransmission of the SNc DA neurons in Ddc^KI^ mice, likely because of a DA deficiency in the striatum. Dopamine D2-receptors on the cell bodies of SNc DA neurons primarily bind to Kir 3.2 (GIRK2)-containing channels, and the activation of GIRK channels leads to potassium efflux, which hyperpolarizes DA cells and impairs firing (Beckstead et al., 2004). D2-mediated inhibition must be lacking in the presence of a DA deficiency.

### Correlation Between the Low AHP and Slow, Irregular Basal Firing Pattern in Ddc^KI^ Mice

We observed a low AHP in the APs of regular basal firing SNc DA neurons in the Ddc^KI^ mice, and gene therapy with AAVN-AADC reversed this abnormality. The opening of small conductance calcium-activated potassium (SK) channels following an AP emission generates AHP, which serves as a feedback mechanism to reduce the AP duration and defines a time-window that is refractory to the emission of a new AP (Faber et al., 2005). Glutamate stimuli decrease AHP by inactivating the SK channel and trigger burst firing from neurons (Park et al., 2010; Paladini and Tepper, 2017). However, pharmacological inhibition of SK channels increases the variability of basal firing (Iyer et al., 2017), and a mutation of the SK channel increases basal firing irregularity (Soden et al., 2013). In the current study, the mRNA expression level of Kcnn3 also decreased (*p* = 0.05).

Interestingly, a reduction in AHP and an increase in irregular firing were also observed in other animal models of DA deficiency, including the *PINK-1* and *HrtA2/Omi*-deficient mouse model of PD (Bishop et al., 2010) and TH-deficient mice (Paladini et al., 2003). Therefore, an increase in glutamatergic excitatory stimulation and a decrease in GABAergic and D2 inhibitory stimuli likely cause over-inhibition of SK channels in the presence of a generalized dopamine deficiency, resulting in irregular firing.

### A Proposed Mechanism for Motor Disturbances

**Figure 6:**
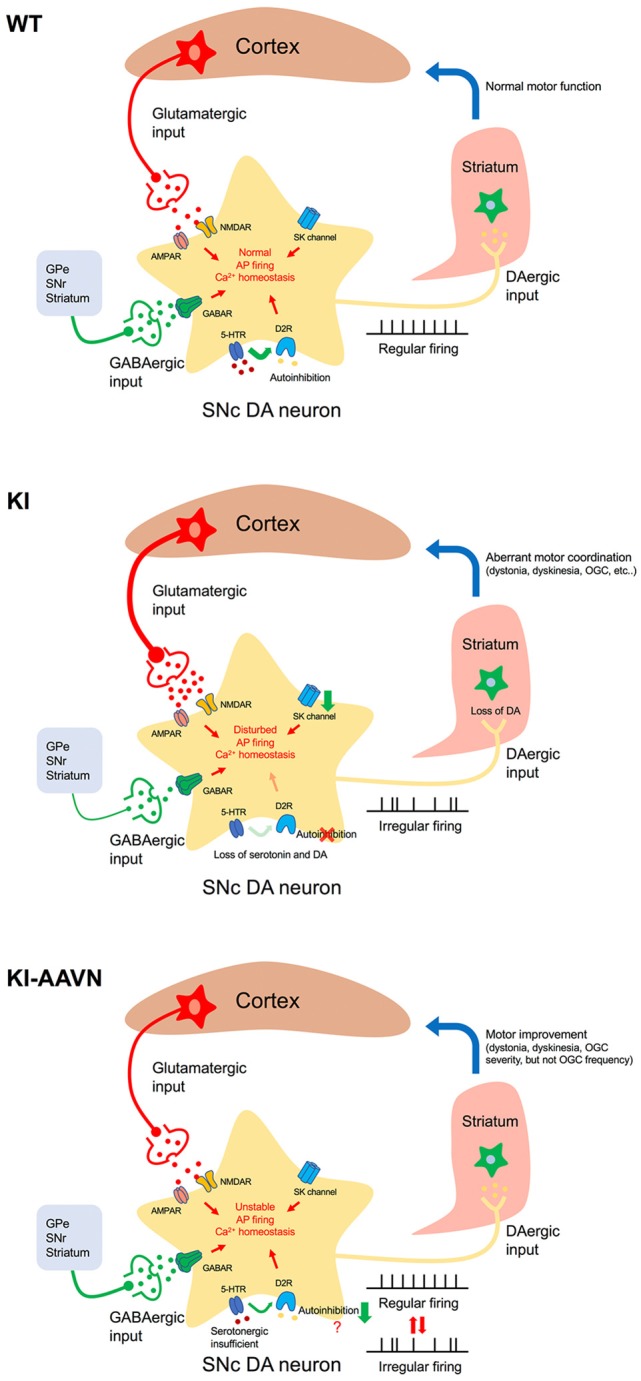
Proposed mechanism of motor disturbances. Excitation/inhibition imbalance, loss of autoinhibition, and SK channels downregulation disturbs cytoplasmic Ca^2+^ homeostasis in AADC deficiency SNc DA neurons (KI). Basal firing slows, the DA deficiency in the putamen worsens, and motor disturbances occur. Gene therapy treatment (KI-AAVN) reverses these electric abnormalities and relieves the symptoms of mice. However, the insufficient levels of serotonin may gradually, and repeatedly, influence the DA neurons and cause irregular firing patterns, which then lead to motor disturbance [rhythmic oculogyric crisis (OGC)].

Tonic DA release in the striatum, which is driven by pacemaking basal firing, is required to maintain the DA levels necessary for motor activity (Goto et al., 2007). A hypothesis for cyclic motor disturbance in patient with AADC deficiency may be explained by low-irregular firing worsens the status of DA deficiency in the putamen in subjects with an AADC deficiency, which leads to the motor disturbances, such as severe dyskinesia and dystonia. In addition, an increase in burst firing caused by excessive glutamatergic stimulation may also contributes to dopamine depletion in the putamen (Figure 6). In the present study, both abnormal firing pattern and the hyperactive glutamatergic signal of DA neuron in Ddc^KI^ mice are significantly decreased after gene therapy. One possible reason for the restoration may be associated with an increment in dopamine level in the brain. Furthermore, we have previously reported that gene therapy is effective and improves motor function in patient and mouse with AADC deficiency (Hwu et al., 2012; Lee et al., 2014, 2015). These results suggest that replenished dopamine level in the brain by gene therapy restored motor function and cellular homeostasis in AADC deficiency.

Interestingly, we have previously noted that a special movement disorder, OGC, showing limited improvement in patients treated with gene therapy. These patients displayed a significantly decrease in the severity of OGC episodes but did not change the recurrence of OGC (Hwu et al., 2012). OGC episodes are composed of repeated movements of the eyes and mouth as well as an increase in dystonia and secretion, and each episode can last for a few hours and recur 3–4 days later (Brun et al., 2010). The etiology for OGC is not clear and proposed mechanisms for OGC include an imbalance between striatal DA and cholinergic tones, hypersensitivity of striatal dopamine receptors, neurodegeneration of striatal interneurons, dysfunction of striatal GABAergic interneurons, and maladaptive synaptic plasticity (Barow et al., 2017). In the current study, we found that decrement of Ddc significantly reduced GABAergic transmission onto SNc DA neurons. Although, we were unable to identify the sources of GABA afferent input to the SNc DA neuron from the brain slice available for our study. Injection of the GABA_A_ agonist into the SNr to inhibit GABA neurons activity result in irrepressible saccadic eye movements in a monkey (Hikosaka and Wurtz, 1985). Therefore, it seems plausible that the downregulation of GABAergic neuron activity in SNr may contribute to the severity of OGC but not frequency. Because the frequency of OGC in patients did not change significantly during the study (Hwu et al., 2012), which we postulated the abnormal firing pattern, hyperactive glutamatergic transmission, and hypoactive GABAergic state should be improved. Other signaling pathways may contribute to the OGC rhythmic repetition. A possible explanation for this is that the insufficient serotonin levels in the brain. We found that despite the serotonin levels also increased in the Ddc^KI^ mice after gene therapy treatment, the levels were still lower (~50%) than those in the WT mice. Serotonin has been extensively studied in the hypothalamic suprachiasmatic nucleus for its essential role in rhythmic neuronal activity regulation (Jiang et al., 2000). In addition, serotonin also plays roles in the regulation of DA neuronal firing activity both in a tonic and phasic manner by cooperating with D2 receptors (Olijslagers et al., 2006). Therefore, the effect of insufficient serotonin levels may induce firing changes in DA neurons. This process seems more gradual; thus we were unable to obtain the changes in acute brain slices electrophysiological recording.

### Limitations of the Study

SNc DA neuron burst firing was not analyzed in the present study because burst firing can only be recorded *in vivo*. However, Ddc^KI^ mice are very fragile, and we were not able to obtain a sufficient number of animals for *in vivo* studies. We are preparing an AADC-deficient mouse model that exhibits better survival, which may be suitable for *in vivo* studies in the future. In this Ddc^KI^ mice model we used had been observed a phenomenon of compensatory elevation of brain dopamine and serotonin level at 8 weeks of age (Lee et al., 2013). To avoid this, we choose mice age at 4 weeks for our study.

## Author Contributions

S-YH, H-HL, W-LH and N-CL designed the research studies, conducted the experiments, acquired and analyzed the data, and wrote the manuscript. Y-HC and L-KT conceived the study design and provided reagents. SM provided the viral vector.

## Conflict of Interest Statement

SM owns equity in a gene therapy company (Gene Therapy Research Institution) that commercializes the use of AAV vectors for gene therapy applications and thus may have a potential conflict of interest to the extent that the work in this manuscript increases the value of these commercial holdings. The remaining authors declare that the research was conducted in the absence of any commercial or financial relationships that could be construed as a potential conflict of interest.

## Supplementary Material

FIGURE S1 DA neurons identification in the SNc. Immunofluorescence staining of a SNc neurons filled with biocytin through the glass recording electrode. The TH staining (green), biocytin (red), and colabeling of TH and biocytin are shown. Scale bar, 50 μm.

FIGURE S2 NMDA-induced bursting in *ex vivo* brain slice. **(A)** The representative traces of spontaneous firing from WT and Ddc^KI^ mice. The WT DA neurons typically displayed higher regular firing activity, and exhibited “bursting” firing pattern after NMDA application. In contrast, the Ddc^KI^ DA neurons fired in a more irregular pattern. Interestingly, the Ddc^KI^ DA neurons showed similar “bursting” firing pattern as observed in WT DA neurons. Scale bar: 20 mV and 5 s. **(B)** Comparison of the coefficient of variation of the interspike interval (CV-ISI). The Ddc^KI^ DA neurons displayed a significantly higher CV-ISI than WT DA neurons. However, no differences were detected in the CV-ISI after NMDA-induced bursting. Data were collected from three neurons in the WT group and two neurons in the KI group. **p* < 0.05; ns, not significant.

TABLE S1 Gene expression in SNc of Ddc^KI^ mice (*n* = 3).
