# Supplementary figures and images for: Electrical Abnormalities in Dopaminergic Neurons of the Substantia Nigra in Mice With an Aromatic L-Amino Acid Decarboxylase Deficiency
Source: Front Cell Neurosci. 2019 Jan 31;13:9. doi: 10.3389/fncel.2019.00009 (PMC6365702; doi:10.3389/fncel.2019.00009)

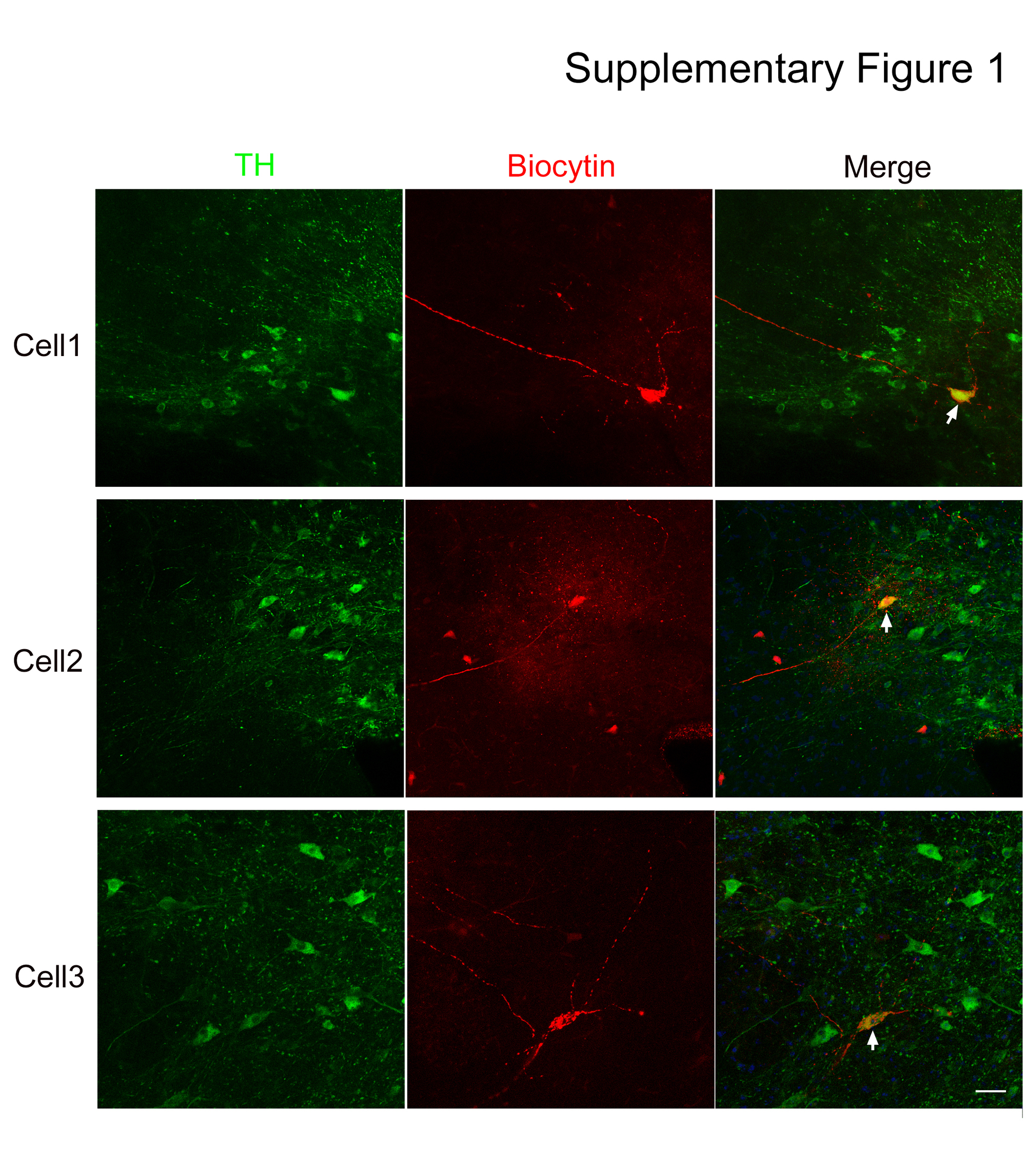

Supplement: FIGURE S1 — DA neurons identification in the SNc. Immunofluorescence staining of a SNc neurons filled with biocytin through the glass recording electrode. The TH staining (green), biocytin (red), and colabeling of TH and biocytin are shown. Scale bar, 50 μm. [file Image_1.jpg]

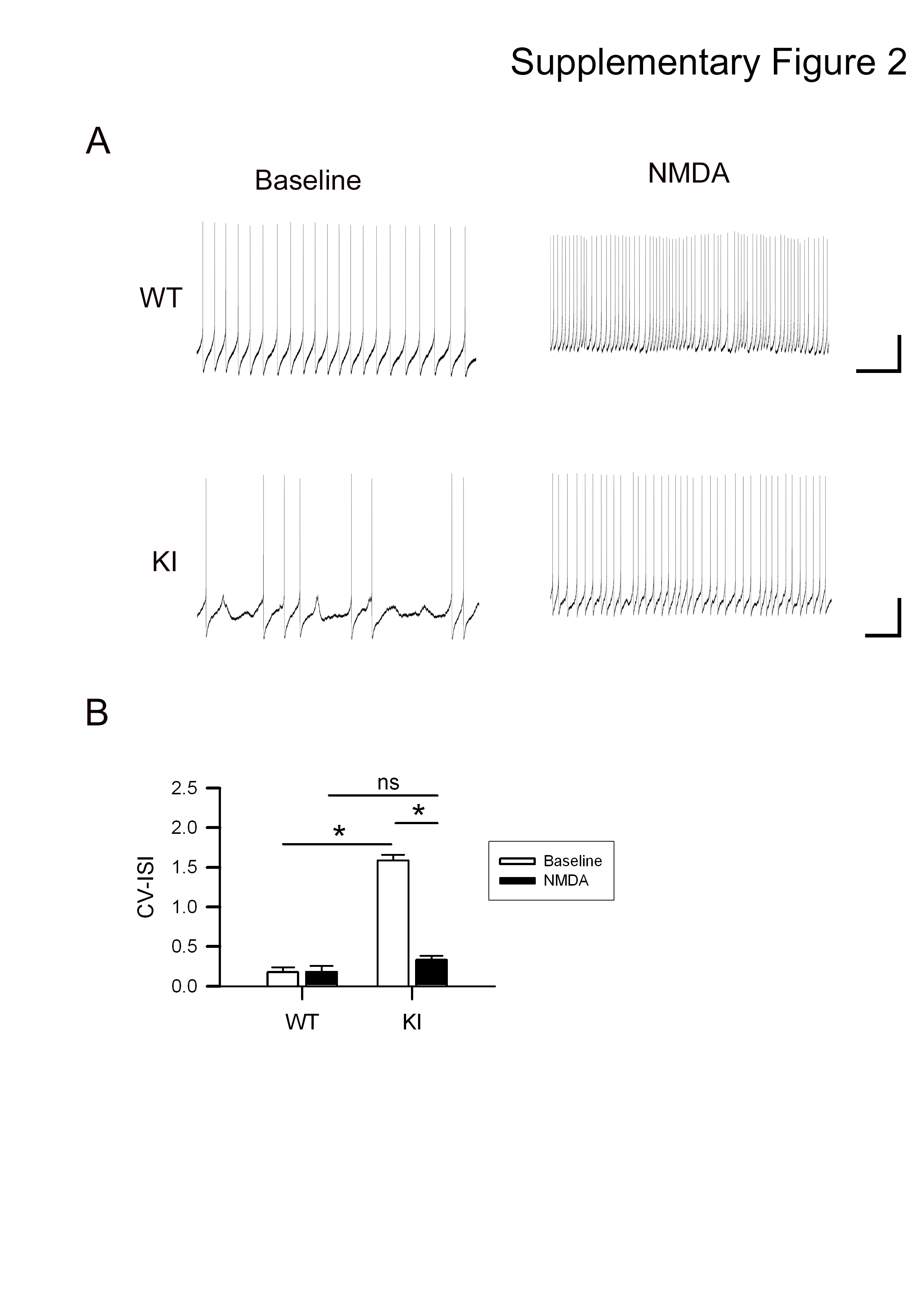

Supplement: FIGURE S2 — NMDA-induced bursting in ex vivo brain slice. (A) The representative traces of spontaneous firing from WT and DdcKI mice. The WT DA neurons typically displayed higher regular firing activity, and exhibited “bursting” firing pattern after NMDA application. In contrast, the DdcKI DA neurons fired in a more irregular pattern. Interestingly, the DdcKI DA neurons showed similar “bursting” firing pattern as observed in WT DA neurons. Scale bar: 20 mV and 5 s. (B) Comparison of the coefficient of variation of the interspike interval (CV-ISI). The DdcKI DA neurons displayed a significantly higher CV-ISI than WT DA neurons. However, no differences were detected in the CV-ISI after NMDA-induced bursting. Data were collected from three neurons in the WT group and two neurons in the KI group. *p < 0.05; ns, not significant. [file Image_2.JPEG]
