# Supplementary material for: Electrical Abnormalities in Dopaminergic Neurons of the Substantia Nigra in Mice With an Aromatic L-Amino Acid Decarboxylase Deficiency
Source: Front Cell Neurosci. 2019 Jan 31;13:9. doi: 10.3389/fncel.2019.00009 (PMC6365702; doi:10.3389/fncel.2019.00009)
Supplement: TABLE S1 — Gene expression in SNc of DdcKI mice (n = 3). [file Table_1.pdf]

Supplemental Table 1. Gene expression in SNc of Ddc^{KI} mice (n=3)

**Category**	$2^{-\Delta CT}$	**WT**		**KI**		**% of WT**	**P**
		**Mean**	**SEM**	**Mean**	**SEM**	$(2^{-\Delta\Delta CT})$	
Dopaminergic neuron	*Th*	18.77	2.93	9.80	0.83	52.20	0.050
	Drd2	1.57	0.31	0.98	0.07	62.30	0.050
Calcium channel	*Cacna1c*	0.68	0.08	1.35	0.44	198.20	0.275
	Cacna1g	0.15	0.03	0.24	0.06	162.90	0.275
Potassium channel	*Kcnj13*	1.41	0.91	0.21	0.04	14.80	0.275
	Kcnq4	0.16	0.05	0.14	0.02	86.90	0.513
	Kcnd3	5.18	0.41	5.08	1.31	98.00	0.827
	Kcnip3	2.54	0.18	1.95	0.32	76.80	0.275
	Kcnn3	0.28	0.03	0.16	0.03	56.80	0.050
	Hcn1	6.19	2.02	6.26	0.76	101.00	0.827
	Grid1	0.00	0.00	0.01	0.00	296.20	0.127
Glutamate receptor	*Grid2*	0.02	0.01	0.04	0.00	194.30	0.050
	Grm1	0.19	0.07	0.50	0.14	264.70	0.127
	Grm5	0.14	0.04	0.26	0.05	185.70	0.275
	Gria1	15.64	2.75	16.19	2.22	103.50	0.827
	Gria2	20.57	3.91	22.47	2.73	109.20	0.513
	Gria3	13.07	2.80	11.71	2.63	89.60	0.513
	Gria4	3.52	0.97	3.91	1.71	111.00	0.827
	Gabbr1	171.31	25.20	167.98	40.32	98.10	0.827
	Gabbr2	3.30	0.89	5.07	1.91	153.90	0.513
GABA receptor	*Gabra3*	2.43	0.53	6.81	2.41	279.90	0.050
	Gabrb1	4.72	1.47	5.60	1.57	118.80	0.275

	Gabrg2	5.09	0.75	7.30	1.44	143.60	0.275
